# The affordable medicines facility-malaria—A success in peril

**DOI:** 10.1186/1475-2875-11-370

**Published:** 2012-11-08

**Authors:** Ambrose O Talisuna, Seraphine Adibaku, Chioma N Amojah, George K Amofah, Vivian Aubyn, Alex Dodoo, Elizabeth Juma, Djermakoye H Jackou, Sigsbert Mkude, Albert P Okui, Benjamin Ramarosandratana, Shija J Shija

**Affiliations:** 1 World Wide Antimalarial Resistance Network (WWARN), Malaria Public Health Department, University of Oxford/KEMRI/Wellcome Trust Research Programme, PO Box 43640, 00100, Nairobi, Kenya; 2 Uganda Malaria Research Centre (UMRC), China Uganda Friendship Hospital, Kampala, Uganda; 3 National Malaria Control Programme, Federal Ministry of Health, Abuja, Nigeria; 4 School of Public Health, Ghana Health Services, Ministry of Health, Accra, Ghana; 5 National Malaria Control Programme, Accra, Ghana; 6 Centre for Tropical Clinical Pharmacology & Therapeutics, University of Ghana Medical School, Accra, Ghana; 7 Kenya Medical Research Institute (KEMRI), Nairobi, Kenya; 8 Programme National de Lutte contre le Paludisme (PNLP - Niger), Niamey, Niger; 9 National Malaria Control Programme (NMCP), Dar es salaam, Tanzania; 10 National Malaria Control Programme, Ministry of Health, Kampala, Uganda; 11 Programme National de Lutte contre le Paludisme (PNLP-Madagascar), Antananarivo, 101, Madagascar; 12 Zanzibar Malaria Control Programme, Ministry of Health-Zanzibar, Zanzibar, Tanzania

**Keywords:** Malaria/economics, Malaria/treatment, AMFm, ACT, Global fund

## Abstract

The Affordable Medicines Facility-malaria (AMFm) has put into place a bold financing plan for artemisinin-combination therapy in a pilot phase in seven countries covering half the population at risk of malaria in Africa. A report of the AMFm independent evaluation, conducted by ICF International and the London School of Hygiene and Tropical Medicine, describes the success of the programme in the pilot sites: Ghana, Kenya, Madagascar, Niger, Nigeria, Tanzania (mainland and Zanzibar) and Uganda, comparing availability and affordability of high-quality artemisinin-combination therapies before and after AMFm launched. Proof of concept was achieved: AMFm increased availability and kept prices low, meeting its initial, ambitious benchmarks in most settings. Despite this overwhelming success, opposition to the programme and dwindling resources for malaria control conspire to cripple or kill AMFm.

## Background

AMFm began with an audacious idea first proposed in a 2004 U.S. Institute of Medicine expert report, *Saving Lives, Buying Time: Economics of Malaria Drugs in an Age of Resistance*[1]. The idea was to subsidize the world’s most effective anti-malarial medicines at the top of the supply chain—at the manufacturers’ factory gates. The drugs would then flow to patients in the same way older, now-ineffective, agents did (and still do). The IOM committee settled on this recommendation to accommodate these realities:

1. ACT, the WHO-recommended first-line medicine for falciparum malaria, is much more costly than their widely-available competitors, now largely ineffectual because of drug resistance.

2. In Africa, anti-malarial medicines are more likely to be purchased from private vendors than from public clinics, which often have stock-outs.

3. Given their general unaffordability, minus a subsidy, many shopkeepers would never stock ACT.

In 2008, the Global Fund to Fight AIDS, Tuberculosis and Malaria assumed responsibility for AMFm in a programme financially and administratively separate from the Fund’s routine grant-making. The high-level subsidy, a vigorous price negotiation conducted by the Clinton Health Access Initiative (CHAI) and a suite of ‘supporting interventions’ were the centerpieces of what was eventually named AMFm.

A pilot involving seven countries was supported by the Roll Back Malaria Partnership, the World Bank and others, including lead funders, the U.K. Department for International Development, UNITAID, and the Bill & Melinda Gates Foundation. Under the AMFm pilot, the first ‘co-paid’ ACT was shipped to Ghana in August 2010 and the rest of the countries came on line over the next eight months.

## Independent evaluation of the AMFm pilot

The Global Fund Board required a thorough evaluation of the pilot that it could rely on to base decisions about AMFm’s future. ICF International and the London School of Hygiene and Tropical Medicine (LSHTM) conducted the evaluation independent of the Global Fund and AMFm. This is the first global public health intervention to be evaluated against stringent benchmarks agreed upon ahead of time [2] and it is among the most thorough and costly such evaluation ever undertaken.

In summary, the evaluation [3,4] reported:

"‘Of the 8 pilots, success benchmarks were clearly met in 5 pilots for availability, 5 pilots for QAACT [quality-assured artemisinin-combination therapy] price relative to the most popular antimalarial that is not a QAACT, and 4 pilots for QAACT market share…. It is also possible that benchmarks were met in one additional pilot for availability and price, and in 3 additional pilots for market share, although the evidence is not as strong…. The success benchmarks related to artemisinin monotherapy (AMT) price and market share were met in all pilots with sufficient AMT in the market to make these benchmarks relevant.’"

AMFm was, in the words of the evaluators, a ‘game changer’ that brought ACT to the village.

### What comes next for AMFm

Because AMFm’s 18-month pilot was largely successful, the global community should now be faced with the happy task of expanding the programme. To wit: AMFm phase 2 would reach even more countries and many more poor and vulnerable people, and ACT would soon be coupled with rapid diagnostic tests (RDTs) to ensure that modern anti-malarial medicines are dispensed only to patients with proven malaria and not those suffering with febrile illnesses from other causes.

Instead, what is looming is that AMFm will either scale down or end altogether. The Global Fund Board is set to vote on AMFm’s future in November 2012, not long before the pilot is set to conclude at the end of 2012. A transition period is planned if AMFm were not to continue though the length of this transition period and the level of support that would be available is not fully clear.

## Discussion

As chloroquine resistance spread around the globe during the end of the 20^th^ century, malaria and child mortality rates increased [5]. Even then, people still used chloroquine because it was available and affordable. ACT was neither available nor affordable in the private sector until AMFm-subsidized packs reached the shelves of shops and pharmacies through the AMFm pilot.

No one denies that defeating malaria requires multiple tools, including insecticide-treated nets, indoor spraying of houses, and other vector control measures. But indisputably, the suite of interventions must also include easily-accessed and effective medicines real people can afford.

It is surprising to malaria control managers in Africa that the donors who generously funded the AMFm pilot would want to move on to other problems. Most health professionals who have been fighting malaria in Africa and have too often suffered from this old scourge agree that money for malaria control must not be wasted. Nor are they blind to other health needs. But AMFm has worked where nothing else does, and even at scale, it should be affordable globally if malaria continues to be prioritized.

The successes of insecticide-treated nets have been trumpeted by several stakeholders [6]. But relative to the problem, the successes are modest. The malaria burden has not, for instance, been halved. If the global community does not persevere with all control measures, well-documented history says the gains made will surely be lost [7]. An important part of continuing to make inroads in the malaria burden is expanding—not contracting—access to high-quality ACT for all who need it. Now, as has been the case for decades, and as the IOM committee observed, the private sector is a complimentary and important supplier of medicines for malaria in most settings in Africa.

## Conclusions

AMFm has proven itself and should be expanded to include more countries and adapted as needed to the changing malaria landscape and country specific context. This may mean finding ways of encouraging the use of RDTs, for instance. But the basic architecture of the AMFm subsidy and price negotiations should continue and expand. The evidence supports AMFm and the cost is not prohibitive for what it delivers. The $300 million spent on the pilot is a mere fraction of the $30 billion or so of the health development aid spent around the world each year [8]. The credibility of the international community in Africa is at stake. AMFm should be a global priority, not merely an interesting footnote of malaria control history.

## Abbreviations

ACT: Artemisinin-combination therapy; AMFm: Affordable Medicines Facility-malaria; AMT: Artemisinin monotherapy; CHAI: Clinton Health Access Initiative; IOM: Institute of Medicine (of the National Academies); LSHTM: London School of Hygiene and Tropical Medicine; QAACT: Quality-assured artemisinin-combination therapy; RDT: Rapid diagnostic test.

## Competing interests

The authors declare that they have no competing interests.

## Author’s contributions

All authors have reviewed and contributed to the content. AOT drafted the commentary for the group. All authors read and approved the final manuscript.
